# Antispasmodic, bronchodilator, vasorelaxant and cardiosuppressant effects of *Buxus papillosa*

**DOI:** 10.1186/s12906-017-1558-x

**Published:** 2017-01-18

**Authors:** Arif-ullah Khan, Shamsher Ali, Anwarul-Hassan Gilani, Manzoor Ahmed, Muhammad Iqbal Choudhary

**Affiliations:** 1 Department of Pharmacology, Riphah Institute of Pharmaceutical Sciences, Riphah International University, Islamabad, Pakistan; 2 Molecular and Integrative Physiological Sciences, Harvard School of Public Health, Boston, MA USA; 3 Department of Biological and Biomedical Sciences, The Aga Khan University Medical College, Karachi, 74800 Pakistan; 4 Pakistan Council of Science and Technology, Islamabad, Pakistan; 5 Department of Chemistry, University of Malakand, Chakdara, Dir (L), Pakistan; 6 International Center for Chemical and Biological Sciences, H EJ Research Institute of Chemistry, University of Karachi, Karachi, Pakistan

**Keywords:** *Buxus papillosa*, Antispasmodic, Bronchodilator, Vasodilator, Cardio-depressant, Ca^++^ antagonist

## Abstract

**Background:**

The present research was carried out to investigate pharmacological properties of *Buxus papillosa* C.K. Schneid. (Buxaceae).

**Methods:**

*Buxus papillosa* extracts of leaves (BpL), stem (BpS), roots (BpR) and BpL fractions: hexane (BpL-H), aqueous (BpL-A) also plant constituent, cyclomicrobuxine effect were studied in jejunum, atria, aorta and tracheal preparations from rabbit and guine-peg.

**Results:**

Ca^++^ antagonistic effect of BpS, BpR, BpL-H, BpL-A and cyclomicrobuxine were conclusively suggested, when spontaneous contractions of rabbit jejunal preparation was relaxed along with subsequent relaxation of potassium chloride (80 mM) induced contractions. Ca^++^ antagonistic effect was further confirmed, when a prominent right shift like that of verapamil was observed in Ca^++^ concentration-response curves, drawn in a tissue pretreated with BpL (0.3–1.0 mg/mL). In rabbit tracheal tissues BpL, BpS, BpR, BpL-H and BpL-A produced a prominent relaxation in contractions induced by potassium chloride (80 mM) and carbachol (1 μm). When tested in rabbit aortic rings, BpL, BpS, BpR, BpL-H and BpL-A showed concentration-dependent (0.1–3.0 mg/mL) vasorelaxant effect against phenylephrine (1 μM) and high K^+^-induced contractions. In isolated guinea-pig right atria, BpL, BpS, BpR, BpL-H and BpL-A suppressed atrial force of spontaneous contractions, with BpL-A being most potent.

**Conclusions:**

Our results reveal that *Buxus papillosa* possesses gut, airways and cardiovascular inhibitory actions.

## Background

The genus Buxus comprises of approximately 70 species commonly called boxwood,which are native to Europe, Asia, Africa, Madagascar, America, Maxico, Caribbean, Cuba and China. *Buxus papillosa* C.K. Schneid, locally known as shamshad is dense, compact ever green shrub of 0.5–1.0 m height, belonging to Buxaceae family. In Pakistan, it is distributed throughout Himalaya regions and northern areas. Traditionally used to cure malaria, rheumatism, skin diseases, headache, also considered useful as antidiarrheal, antisecretorycardiotonic and neurotonicagent [1]. It has been widelyevaluated to unfold its phytochemical profile. *Buxuspappilosa* is known to containcyclobuxupaline-C, cyclopapilosine-*D*, (+)-buxamine-*C*, desoxy-16-buxidienine [2], harappamine [3], moenjodaramine [4], papilicine [5], buxaminol-G,cyclobuxaviridine, papilamine, papilinie [6–8], (+) -buxabenzamidienine, (+)-16α-acetoxybuxabenzamidienine, (+)-buxotrienine, (−)-buxanoldine, (+)-buxanaldinine [9], N-formylcyclomicrobuxeine [10], (+)-buxabenzamidine, (+)-homobuxaquamarine, (+)-norcyclomicrobuxeine, (+)-buxupapine, (+)-Nb-norbuxupapine, cyclobuxoviridine [11], (+)-N-acetyl-N-demethylcyclomicrobuxeine, buxaminone [12, 13], (E)-cyclobuxaphylamine, (Z)-cyclobuxaphylamine, (+)-formylharappamine, (+)-N-formylpapalicine [14, 15], buxatenone, cyclobuxaviramine [16], buxapapilinine [17], (+)papillotrienine, (+)-N(b)-demethylpapillotrienine,(+)-N(b)-demethylharappamine [18], buxapapillosine [19], 11-hydroxybucatenone and buxahejrine [20]. Three alkaloids (Triterpenoids): buxahejramine [(20S) - 20-dimethylamino- 2′-hydroxy- 3beta-methyl-3′-methyl- pentanoylamino-9, 10-seco-buxa-9(11), 10(19)-dien-31-ol], buxakashmiramine [(20S)- 20-dimethylamino-4′,6′- dimethoxy-5′- hydroxybenzoylamino- 3beta-methyl-buxan-31-ol] and buxakashmiramine [(20S)- 20-dimethylamino-4′,6′- dimethoxy-5′- hydroxybenzoylamino- 3beta-methyl-buxan-31-ol] isolated from leaves of *Buxuspapillosa* are reported to possess acetylcholinesteraseand butyrylcholinesterase inhibitory properties [21]. *Buxus papillosa* showed acaricidal effect in larval immersion test [22].

Despite of diverse ethnomedicinal and phytochemical profile of this plant, extensive pharmacological screening has not been performed yet. In our previous studies, we observed that several medicinal plants, which exhibits similar nature of constituents (as present in *Buxus papillosa*) with therapeutic potential in treating ailments of gut, airways, heart and vascular hyperactivity, usually possess spasmolytic properties, occurred via either alone or combinations of different pharmacodynamics mechanisms, like inhibition of Ca^++^ ingress and phosphodiesterase enzyme(s), antagonism of muscarinic receptors and potassium channel activation [23–27]. In the current investigation, the gastrointestinal, respiratory and cardiovascular inhibitory actions of *Buxus papillosa* leaves, stem and roots extracts are reported, also further the leaves extract various fractions: hexane and aqueous. The pure compound, isolated from leaves ofthe plant, cyclomicrobuxine showed antispasmolytic effect.

## Methods

### Plant material extraction, fractionation and isolation

**Fig. 1:**
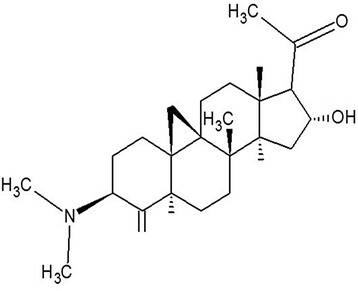
Chemical structure of cyclomicrobuxine, pure compound isolated from leaves of *Buxus papillosa*

*Buxus papillosa* was collected from Malam Jabba, Swat, Khyber Pakhtunkhwa (KPK), Pakistan during July-August, 2002. Specimen was authenticated by Mahboob-ur-Rahman, taxonomist at Department of Botany, Government Post Graduate Jahanzeb College, Saidu Sharif, Swat, KPK. Voucher bearing number BP-129 was obtained and specimen submitted to herbarium at same college for future record. Plant material was dried under shade for 20-25days and separated into leaves (44.7 Kg), stem (14.5 Kg) and roots (42 Kg) parts. All three parts were individually ground and macerated in aqueous methanol (70%) for period of 7 days at room temperature [28, 29]. Mixtures were then filtered and concentrated under reduced pressure to yield*Buxuspappilosa*extracts of leaves (BpL), stem (BpS) and roots (BpR). For defatting the extract of leaves, distilled water was added and mixed with it thoroughly, followed by mixing with *n*-hex in a separating funnel. When two distinct layers were formed the *n*-hexane portion was separated out and the whole process was repeated thrice. Finally, the entire collected *n-*hexane portion was concentrated in rotary to receive (BpL-A). Similarly for obtaining Aqueous fraction (BpL-A), the lower layer in the separating funnel was evaporated in separate Petri dish. Cyclomicrobuxine (Fig. 1), a known compound [30, 31] was isolated from hexane fraction of *Buxuspapillosa*leaves extract, using silica gel column chromatography technique [32].

### Chemicals

Acetylcholine chloride (ACh), verapamil, isoprenaline hydrochloride, carbachol (CCh) and phenylephrine hydrochloride (PE) were purchased from Sigma Chemicals Co. MO, USA. Various kinds of chemical were used for the preparation of physiological solutions like: magnesium sulfate,sodium dihydrogen phosphate, calcium chloride, glucose, sodium bicarbonate ,magnesium chloride and potassium dihydrogen phosphate were purchased from Merck, Germany, while potassium chloride (KCl), ethylenediamine tetraacetic acid from Sigma Chemical Co, USA. Similarly Sodium chloride was purchased from BDH Laboratory, England. Analytical grade chemical were used for performing the experiments.

### Animals

For conducting this study Adult guinea-pigs (500–550 g) and rabbits (1–1.2 kg) were collected from the Animal House of Aga Khan University. Standard diet and water were given to all of them under maintained temperature of 23–25 °C. Rabbits and guinea-pigs were starved for 24 h before starting the experiments and were scarified by cervical dislocation to obtain the target tissue. Studies conducted completely comply with the protocols of the Institute of Laboratory Animal Resources, Commission on Life Sciences, National Research Council [33], approved by Research and Ethics Committee (Ref#: REC/RIPS/2016/005), Riphah Institute of Pharmaceutical Sciences.

### Rabbit jejunum

Three segments of jejunum, each 2 cm were dissected out from rabbit abdomen. Single segment of jejunum was suspended via a thread in a tissue bath continuously supplied with carbogen. Tyrode’s solution used is composed of (mM): NaHCO_3_ 11.90, glucose 5.55, CaCl_2_ 1.8, KCl 2.68, MgCl_2_ 1.05, NaCl 136.9 and NaH_2_PO_4_ 0.42. Bioscience transducers and Harvard oscillograph was used for recording intestinal responses isotonically. Before addition of drug, tissue was freely permitted to equilibrate for 30 min. The tissue was then completely stabilized by applying sub-maximal concentration of 0.3 μM Ach after every three minutes, unless uniform responses were achieved. Spontaneous contractions are exhibited in such environment, thereby providing an opportunity to directly check the spasmolytic effect, without prior applying of agonist [34]. Depolarization of jejunal preparations was done by high K^+^ (80 mM), so that Ca^++^ channel blockade (CCB) activity can be determined according to procedure of Gilani et al. [35]. After sustained contractions produced by high K^+^ in jejunal tissue, test substance was applied for producing inhibitory effect. For confirming the Ca^++^ antagonistic activity, the jejunal tissue was stabilized in normal and Ca^++^-free Tyrode’s solutions. After 30 min the tissue was washed with by K^+^-rich and Ca^++^-free Tyrode’s solution. CRCs of Ca^++^ were drawn and recorded. The treated tissue was applied with test substance and incubated for one hour, followed by reconstruction of CRCs of Ca^++^ to explore the possible CCB effect. Different concentration of test substance was applied on treated tissue and CRCs of Ca^++^ were constructed.

### Rabbit trachea

Soon after dissection of trachea, approximately 2–3 mm wide tracheal tube rings were cut down, each ring containing two cartilages were opened further by longitudinal incision to prepare tracheal strips [36]. Single tracheal strip was suspended in tissue bath (20 mL) containing standard physiological kreb’s solution, continuously supplied with carbogen and maintained at 37 °C (pH 7.4). Kreb’s solution used for this experiment was composed of (mM) glucose: 11.7, KH_2_PO_4_: 1.2, NaCl: 118.2, CaCl_2_: 2.5, MgSO_4_. 7H_2_O: 1.2, NaHCO3: 25.0, KCl: 4.7. Strips were permitted to equilibrate for almost 1 h before applying any test substance. Throughout experiment tracheal strips were applied with tension of 1 g. After the application of spasmogens like CCh and/or high K^+^, when sustained contractions were achieved then spasmolytic effect of drug was tested by its addition in cumulative manner. Grass model 7 Polygraph (USA) was used for recording the isometric responses of tracheal strips.

### Rabbit aorta

Krebs solution in a 20 mL tissue bath was used for conducting the experiment; aortic rings were mounted in it, where bath environment was maintained at 37 °C along with continuous supply of carbogen. Before studying the effect of drug, aortic ring was stabilized by applying tension of 2 g. After the application of PE (1 μM) and K^+^ (80 mM), vasorelaxant effect of testing material was assessed [37]. Force-displacement transducer and Grass model 7 Polygraph were used for recording changes in isometric tension of aortic rings.

### Guinea-pig atria

Right atrium was isolated and suspended through wire gauze in 20 mL tissue bath containing Krebs solute, maintained at 32 °C continuously supplied with carbogen gas. Due to presence of natural pacemaker in the atrium, Spontaneous beating was observed under the resting tension of 1 g [38]. After 45 min of equilibrium period, control response curves of 1 μM, isoprenaline and ACh were recorded. Force-displacement transducer and Grass model 7 Polygraph were used for recording changes in isometric tension of atria.

### Statistical analysis

Recorded data obtained is shown as mean ± standard error of mean (SEM, n = number of experiment) and median effective concentrations (EC_50_) with 95% confidence intervals (CI). GraphPad program (USA) was used by applying non-linear regression for analyzing Concentration-response curves.

## Results

### Effect on jejunum

**Fig. 2:**
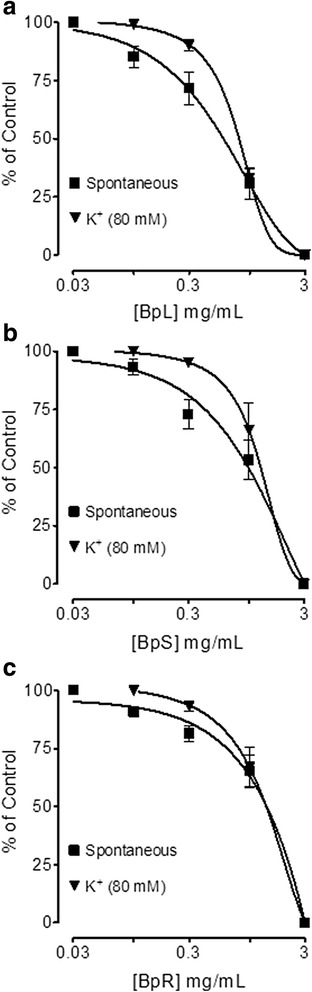
Concentration-dependent inhibitory effect of *Buxus papillosa* different parts extracts: **a** leaves (BpL), **b** stem (BpS) and **c** roots (BpR) on spontaneous and high K^+^-induced contractions in isolated rabbit jejunum preparations. Values shown are mean ± SEM, *n* = 4–6

**Fig. 3:**
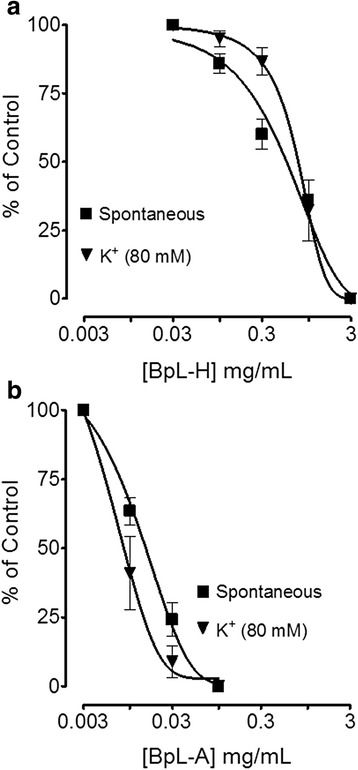
Concentration-dependent inhibitory effect of *Buxus papillosa* leaves extract fractions: **a** hexane (BpL-H) and **b** aqueous (BpL-A) on spontaneous and high K^+^-induced contractions in isolated rabbit jejunum preparations. Values shown are mean ± SEM, *n* = 3–6

**Fig. 4:**
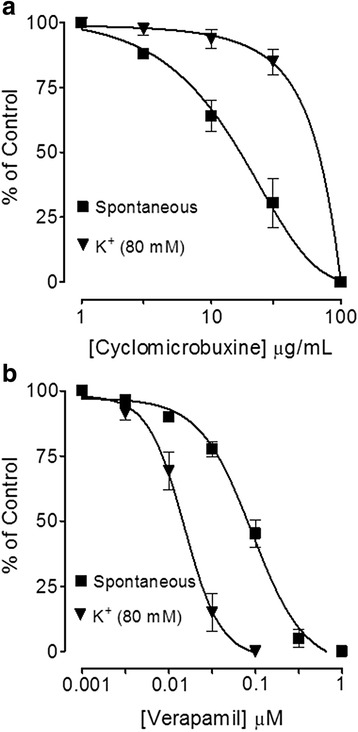
Concentration-dependent inhibitory effect of **a** cyclomicrobuxine, a constituent of *Buxux papillosa* and **b** verapamil on spontaneous and high K^+^-induced contractions in isolated rabbit jejunum preparations. Values shown are mean ± SEM, *n* = 2–4

**Fig. 5:**
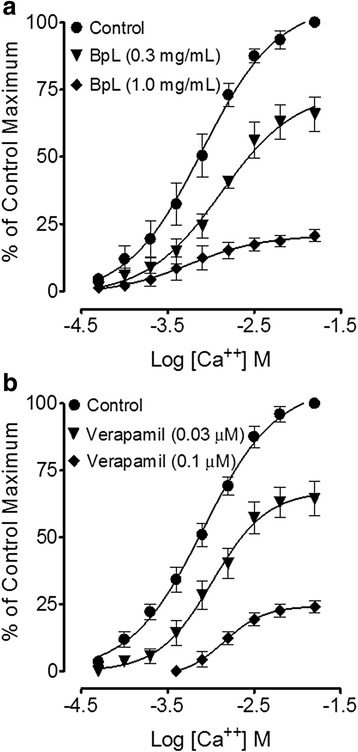
Concentration-response curves of Ca^++^ in absence and presence of increasing concentrations of **a** *Buxus papillosa* leaves extract (BpL) and **b** verapamil in isolated rabbit jejunum preparations. Values shown are mean ± SEM, *n* = 3–4

Isolated jejunal preparations spontaneous contractions and induced contractions of high K^+^ (80 mM) were concentration dependently inhibited by BpL, BpS, BpR, BpL-H and BpL-A with respective EC_50_ values of 0.63 (0.46–0.86, *n* = 5, 95% CI) and 0.80 (0.71–0.84, *n* = 6), 1.02 (0.70–1.50, *n* = 5) and 1.20 (0.85–1.65, *n* = 4), 1.25 (0.99–1.65, *n* = 6) and 1.23 (0.94–1.54, *n* = 5), 0.55 (0.42–0.74, *n* = 3) and 0.73 (0.62–0.89, *n* = 3), 0.014 (0.008–0.02, *n* = 6) and 0.008 mg/mL (0.001–0.04, *n* = 4) as shown in Figs. 2, 3 and Table 1. Cyclomicrobuxine relaxed spontaneous contractions and induced contractions of high K^+^ (80 mM) with EC_50_ values of 21.0 (10.1–30.3, *n* = 2) and 65.1 μg/mL (50.1–70.6, *n* = 2) respectively (Fig. 4a). Verapamil relaxed spontaneous and potassium chloride (80 mM)-induced contractions (Fig. 4b) with respective EC_50_ values of 0.09 (0.07–0.11, *n* = 4) and 0.013 μM (0.01–0.02, *n* = 4). BpL (0.3–1.0 mg/mL) shifted Ca^++^ CRCs to the right (Fig. 5a), like that caused by verapamil (Fig. 5b).

**Table 1:** Comparative median effective concentration (EC_50_) values of the *Buxus papillosa* different parts extracts: leaves (BpL), stem (BpS) and roots (BpR) also leaves extract fractions: hexane (BpL-H) and aqueous (BpL-A) against various parameters in different isolated tissue preparations of rabbit and guinea-pig (atria)

Sample	Jejunum		Trachea		Aorta		Atria
	Spontaneous	K^+^ (80 mM)	CCh (1 μM)	K^+^ (80 mM)	PE (1 μM)	K^+^ (80 mM)	Atrial force
BpL	0.63	0.80	0.28	0.28	0.75	0.50	4.0
BpS	1.02	1.20	0.26	0.85	0.98	1.3	1.80
BpR	1.25	1.23	0.10	0.34	1.25	0.95	3.60
BpL-H	0.55	0.73	0.25	0.31	0.98	1.1	1.50
BpL-A	0.014	0.008	0.12	0.10	0.40	0.37	0.30

### Effect on trachea

**Fig. 6:**
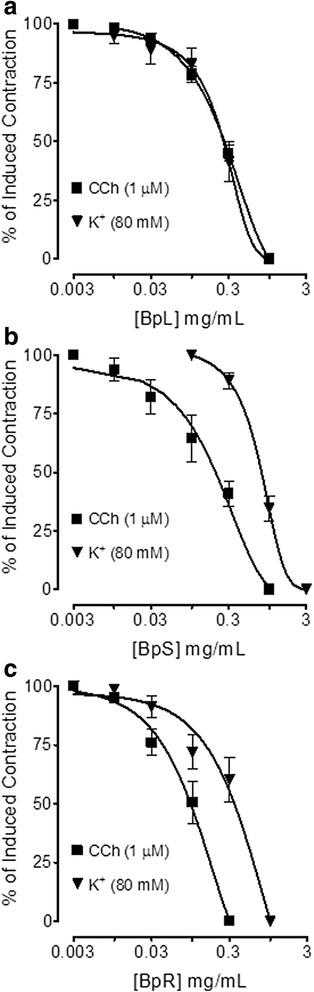
Concentration-dependent inhibitory effect of *Buxus papillosa* different parts extracts: **a** leaves (BpL), **b** stem (BpS) and **c** roots (BpR) on carbachol (CCh) and high K^+^-induced contractions in isolated rabbit tracheal preparations. Values shown are mean ± SEM, *n* = 4–5

**Fig. 7:**
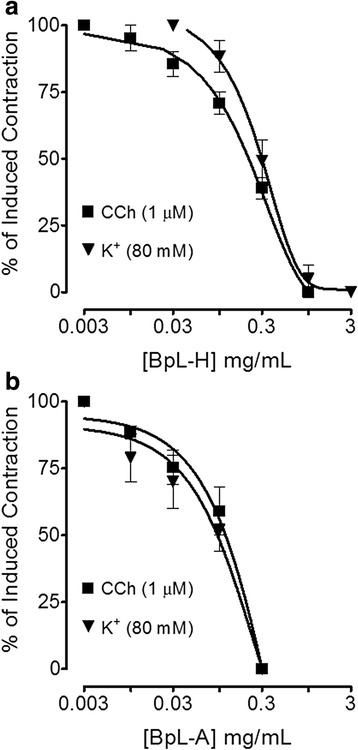
Concentration-dependent inhibitory effect of *Buxus papillosa* leaves extract fractions: **a** hexane (BpL-H) and **b** aqueous (BpL-A) on carbachol (CCh) and high K^+^-induced contractions in isolated rabbit tracheal preparations. Values shown are mean ± SEM, *n* = 2–3

Pre-contracted, tracheal preparations with CCh (1 μM) and K^+^ (80 mM) were concentration-dependently relaxed by BpL, BpS, BpR, BpL-H and BpL-A with EC_50_ values of 0.28 (0.24–0.33, *n* = 5) and 0.28 (0.22–0.36, *n* = 4), 0.26 (0.13–0.41, *n* = 4) and 0.85 (0.70–0.94, *n* = 4), 0.10 (0.07–0.13, *n* = 4) and 0.34 (0.23–0.59, *n* = 4), 0.25 (0.16–0.33, *n* = 2) and 0.31 (0.27–0.37, *n* = 2), 0.12 (0.08–0.19, *n* = 2) and 0.10 mg/mL (0.04–0.25, *n* = 3) respectively (Figs. 6, 7 and Table 1).

### Effect on aorta

**Fig. 8:**
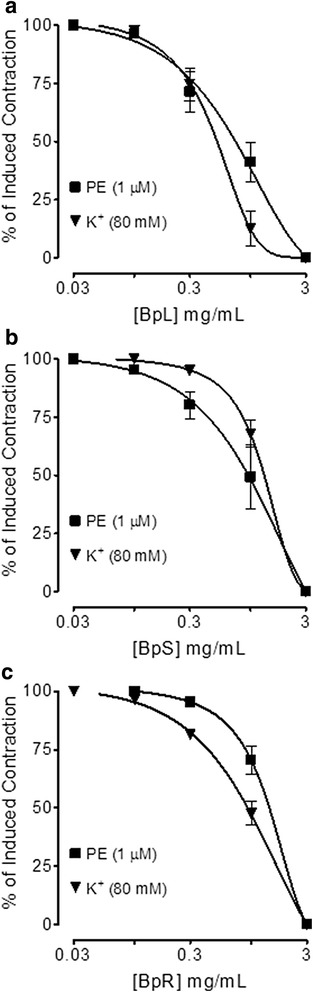
Concentration-dependent inhibitory effect of *Buxus papillosa* different parts extracts: **a** leaves (BpL), **b** stem (BpS) and **c** roots (BpR) on phenylephrine (PE) and high K^+^-induced contractions in isolated rabbit aortic preparations. Values shown are mean ± SEM, *n* = 4–5

**Fig. 9:**
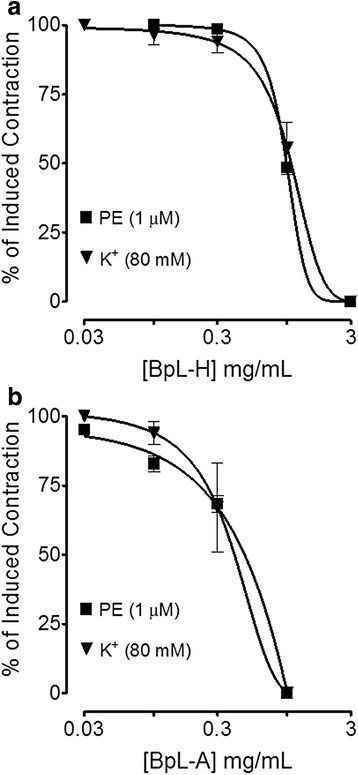
Concentration-dependent inhibitory effect of *Buxus papillosa* leaves extract fractions: **a** hexane (BpL-H) and **b** aqueous (BpL-A) on phenylephrine (PE) and high K^+^-induced contractions in isolated rabbit aortic preparations. Values shown are mean ± SEM, *n* = 2–3

When tested against induced contractions of potassium chloride (80 mM) and PE (1 μM), BpL, BpS, BpR, BpL-H and BpL-A exhibited vasorelaxant effect (Figs. 8, 9 and Table 1) with respective EC_50_ values of 0.75 (0.50–1.1, *n* = 4) and 0.50 (0.40–0.60, *n* = 5), 0.98 (0.70–1.41, *n* = 4) and 1.3 (1.0–1.50, *n* = 4), 1.25 (0.91–1.61, *n* = 4) and 0.95 (0.81–1.11, *n* = 4), 0.98 (0.96–1.0, *n* = 3) and 1.1 (0.92–1.3, *n* = 2), 0.40 (0.23–0.71, *n* = 2) and 0.37 mg/mL (0.16–0.89, *n* = 2).

### Effect on atria

**Fig. 10:**
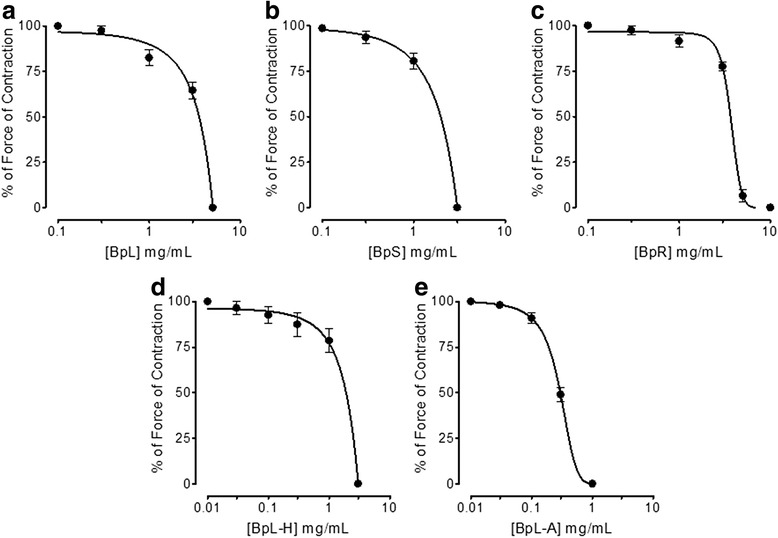
Concentration-dependent inhibitory effect of *Buxus papillosa* different parts extracts: **a** leaves (BpL), **b** stem (BpS) and **c** roots (BpR) also leaves extract fractions: **d** hexane (BpL-H) and **e** aqueous (BpL-A) on force of contraction of the spontaneously beating isolated guinea-pig right atrial preparations. Values shown are mean ± SEM, *n* = 3

BpL, BpS, BpR, BpL-H and BpL-A exerted concentration-dependent inhibitory effect on spontaneously beating atrial force of contractions with EC_50_ values of 4.0 (3.0–5.5, *n* = 3), 1.80 (0.25–6.30, *n* = 3), 3.60 (3.50–4.0, *n* = 3), 1.50 (0.44–4.0, *n* = 3) and 0.30 mg/mL (0.28–0.35, *n* = 3) respectively (Fig. 10 and Table 1).

## Discussion

When tested in jejunum preparations, *Buxus papillosa* different parts extracts and fractions exhibited antispasmodic effect through inhibition of spontaneous contractions. Antispasmodic effect of aqueous fraction was 45 and 40 times more potent as compared to parent leaves extract and hexane fraction respectively. The plant isolated compound, cyclomicrobuxine also relaxed jejunum spontaneous contractions. When the level of free [Ca^++^] increases the rudiments responsible for contractions in the cells get activated, leading to ultimate contraction of smooth muscles [39, 40]. Intracellular calcium level either increases through voltage dependent L-type Ca^++^ channels (VDCs) or through discharge from sarcoplasmic reticulum internal stores. Regulation of Spontaneous intestinal contractions is primarily attributed to intermittent depolarization and action potential appears at the height of depolarization when rapid influx of Ca^++^ via VDCs takes place [41]. So the spontaneous intestinal contractions may be inhibited by the plant extract due to interruption with Ca^++^ release inside the cell or Ca^++^ influx through VDCs.

Previous studies strongly suggest that plant extracts usually mediate relaxation through blockade of Ca^++^ channels [42–46]. To check out whether same mechanism is followed by this plant extract for achieving spasmolytic effect, *Buxus papillosa* test samples were screened against contractions induced by potassium chloride (80 mM) which was fully inhibited, with Aqueous fraction being most potent. Plant bioactive constituent, cyclomicrobuxine also relaxed contractions induced by potassium chloride (80 mM). It was observed that, cyclomicrobuxine was 30 and 12 times more potent respectively in comparison to plant extracts, against spontaneous and potassium chloride induced contractions. Verapamil, a standard Ca^++^ channel blocker [47] also inhibited spontaneous contractions and contractions induced by potassium chloride (80 mM) non-specifically. Smooth muscle contraction is induced by high concentration of K^+^ (>30 mM) via opening of VDCs, where it permits entrance of Ca^++^ from outside the cell [48]. So, drug inhibiting potassium chloride (80 mM) induced contractions is believed to be the inhibitor of Ca^++^ influx [49]. When plant extract exhibited right shift in the Ca^++^ CRCs and suppressed the maximal response like that of a standard calcium antagonist; verapamil, the existence of Ca^++^ antagonist effect strengthened. Concentration-dependent inhibition of slow entry of Ca^++^ is one of the prominent and common features of calcium antagonists [50, 51]. *Buxus papillosa* has been medicinally used in gut hyper motility disorder and diarrhea. Ca^++^ channel blockers are usually effective in this condition [52] and the observed CCB effect of this plant may clarify its potential in these diseases.

In trachea, *Buxus papillosa* test samples relaxed the contractions induced by high K^+^ and CCh, showing tracheo-relaxant effect. Smooth muscles contractions is produced by cholinergic agonist like CCh and high K^+^ via stimulation of muscarinic receptor and opening of L-type Ca^++^ channel respectively, finally enhancing Ca^++^ level inside the cell and bronco constriction as the ultimate result [53]. Non-specific inhibition of two spasmogens by the plant extract suggest nonspecific bronchodilatory effect, probably mediated through calcium channel blockade mechanism. For relieving symptoms of congestive respiratory disorders Ca^++^ antagonists are efficient [54] and existence of CCB activity in B*uxus papillosa* as revealed by this study may provide basis for this plant as an effective agent for treating asthma.

The plant *Buxuspapillosa* was also studied in vascular and heart tissues for possible cardio-depressent and vasorelaxant actions, due to well known efficacy of Ca^++^ antagonists in cardiovascular disorders, such as hypertension [55, 56]. When applied on isolated aorta preparations, *Buxus papillosa* material inhibited contractions produced by high K^+^ and PE. Vascular contraction is induced by PE via increase in cytosolic Ca^++^, to a degree because of Ca^++^ entry via receptor operated channels and up to some extent due to Ca^++^ release from intracellular stores [57]. Non-specific inhibition of high K^+^ and PE by the plant extract suggests nonspecific vasodilatory effect, probably mediated via CCB mechanism. The product of cardiac output and vascular resistance results blood pressure [58], so the likely inhibitory effect of this extract on heart was studied. Atrial force of contractions was inhibited by *Buxus papillosa* in spontaneously beating atria of guinea-peg.

Among all tested samples of the plant, aqueous fraction most potently caused cardiac depression, being 13 times more potent than parent extract. Cardiac inhibitory effect of *Buxus papillosa*, occurred possibly via CCB pathway (observed in gut, trachea and vascular tissues experiments), as Ca^++^ antagonists reportedly possess negative inotropic and chronotropic effects [59]. All tested samples of *Buxus papillosa* (except aqueous fraction, which was about 10 times more potent in relaxing intestinal preparations than tracheal) were relatively more powerful spasmolytic in airways, in comparison to other targeted tissues, this might be due to heterogenicity of Ca^++^ channels, as known to be heterogeneous [60, 61]. Various types of Ca^++^ channels blockers, for different organ system are reported to demonstrate selectivity [62, 63]. For instance, nifedipine reportedly is a vascular selective, as compared to heart [64]. On the other hand, there is also the possibility of phosphodiestarse inhibitors (PDEIs)-like constituent(s), which have impaired cardiac inhibitory potency. We have reported co-existence of PDEIs component(s) with Ca^++^ antagonist(s) in some medicinal plants [65–68]. As, combination of PDEIs and Ca^++^ antagonists have synergistic spasmolytic interaction in smooth muscles (more effective in respiratory system) and opposing/side effects neutralizing effects in heart [69], hence might making the plant more efficacious to relax airways, compared to cardiac muscles.

## Conclusions

These results reveal that *Buxus papillosa* possesses antispasmodic, bronchodilatory, vasodilator and cardiac inhibitory effects. Thus, this study provides scientific evidence for its potential therapeutic application in hyperactive gastrointestinal, respiratory and cardiovascular disorders.

## Abbreviations

ACh: Acetylcholine chloride
BpL: *Buxuspapillosa*leaves extract
BpL-A: *Buxuspapillosa*leaves extract aqueous fraction
BpL-H: *Buxuspapillosa*leaves extract hexane fraction
BpR: *Buxuspapillosa*root extract
BpS: *Buxuspapillosa*stem extract
CCB: Ca^++^ channel blockade
CCh: Carbachol
KPK: Khyber Pakhtunkhwa
PDEIs: Phosphodiestarse inhibitors
PE: Phenylephrine hydrochloride
VDCs: Voltage dependent L-type Ca^++^ channels
